# Supplementary material for: Identification of Seven Additional Genome Segments of Grapevine-Associated Jivivirus 1
Source: Viruses. 2022 Dec 22;15(1):39. doi: 10.3390/v15010039 (PMC9862270; doi:10.3390/v15010039)
Supplement: Supplementary file 1 [file viruses-15-00039-s001.zip › Supplementary Figures-V1.pdf]

Table S1. Primers used for the detection or the molecular characterization of the isolates of GaJV1 in the present study

Primer name	Sequence 5'-3'	Genome positions	Amplicon size (bp)	Annealing T°
**RACE primers**				
GaJV-1 RNA1-3'Race	TGTTCCTTGTYAAACCCGATCACGTGGC	3523-3550	550	70°C
GaJV-1 RNA2-3'Race	GTGTCYTTTTCGGATCGCGTGCGTTCC	2987-3013	244	70°C
GaJV-1 RNA3-3'Race	CGCAACTTACGTATGATYTGGATTGGCC	1738-1765	402	70°C
GaJV-1 RNA4-3'Race	CCGCTTCTTTGCGTAAATTGCTGTCACG	1852-1879	158	70°C
GaJV-1 RNA5-3'Race	TGTTGGGCGTGGCTCTGCTTGGTCGCG	1642-1668	220	70°C
GaJV-1 RNA6-3'Race	GAAGGTGTGTTAGTGTGGAATTCTGTCG	1370-1397	294	70°C
GaJV-1 RNA7-3'Race	CGAAGGTCCCGATTTTGATTCTGTGCG	1175-1201	475	70°C
GaJV-1 RNA8-3'Race	AAGCTTGGTGGGGTCAATAATTGGTCGCG	951-979	912	70°C
GaJV-1 RNA9-3'Race	CTGCTGGCGCTTCTGATCGTCTTATGC	1373-1347	308	70°C
GaJV-1 RNA1-5'Race	CATCTTGGAATCAACAACATGAGGTACC	353-326	353	70°C
GaJV-1 RNA2-5'Race	GCAACATCTCAGCAAATTCAGCAGTACTA	179-151	179	70°C
GaJV-1 RNA3-5'Race	CGAGCATCTTGTGTCTGAACCTCTATGG	256-229	256	70°C
GaJV-1 RNA4-5'Race	CAAACTTCGAATCATCAACAACACAGGCC	196-168	196	70°C
GaJV-1 RNA5-5'Race	CCATGCGCGCAAAACCAAAACCCGAACG	428-401	428	70°C
GaJV-1 RNA6-5'Race	CGAGCTGACAAAACCGGAACGTTGGTGG	462-435	462	70°C
GaJV-1 RNA7-5'Race	CCCAGTAGGTGAACTCAATAAAGAGGAC	353-326	353	70°C
GaJV-1 RNA8-5'Race	CGCAAGAATTAGGCTTAATGCAAGAAA	270-244	270	70°C
GaJV-1 RNA9-5'Race	CTATCTCCAAACACGTTGACTTCATCGGG	300-272	300	70°C
**Gap filling primers**				
CVJ-i31-RNA5-trou-F	CTTTCCAGTGAAGGCTGGCG	942-961	864	60°C
CVJ-i31-RNA5-trou-R	TTTAATCGTCAACCGGAGAAAAC	1806-1787		
3'polyA anchored PCR				
LD-polyT	CACTGGCGGCCGCTCGAGCATGTACT_{25}NN			70°C
LD-prim	CACTGGCGGCCGCTCGAGCATGTAC			
**Detection primers**				
RNA1-CVJ-likeF	GGAATCAAATCTATGGAAGTTG	1191-1212	565	56°C
RNA1-CVJ-likeR	CCGAAAGATCATGCAAATCACC	1755-1734		
RNA2-CVJ-likeF	TGTTCGTTGCCCTTCCCTTG	1201-1220	747	56°C
RNA2-CVJ-likeR	GCATCCAGCAAAGGTGTGTTA	1947-1927		
RNA3-CVJ-likeF	ACGTTGGAGGATGCGAAAGG	169-188	269	56°C
RNA3-CVJ-likeR	CCAAGCATCAAACGGTGACG	437-418		
RNA4-CVJ-likeF	CCGGTTTGTGCTATGCTCG	530-548	346	56°C
RNA4-CVJ-likeR	CCAGACGCACCACCAATACA	875-856		
RNA5-CVJ-likeF	GGTATGCATTCGTCTAGGTC	250-269	898	56°C
RNA5-CVJ-likeR	CCTCCTTCCAAACCAACGCT	1147-1128		
RNA6-CVJ-likeF	GTTTGCGTGTTCGTGCTCC	357-375	486	56°C
RNA6-CVJ-likeR	GTCCTCGCTTAACATCTTCGT	842-822		
RNA7-CVJ-likeF	GTTCTATGGGTAAGGAAATGTC	757-778	444	56°C
RNA7-CVJ-likeR	GCACAGAATCAAAATCGGGAC	1200-1180		
RNA8-CVJ-likeF	CTGGTGGTGTGTCGTCGG	439-456	199	56°C
RNA8-CVJ-likeR	CACAAGCCGTCTTCAACAGG	637-618		
RNA9-CVJ-likeF	TAAGGCCGGTGTATTCGTGC	328-347	429	56°C
RNA9-CVJ-likeR	GCACCAACAACACGCTTACG	756-737		

A)

```
RNA1     ----------------CACCTACCCAAACGAAAC-----GAAACGAAACGAAACGAAACGAAAACAAAAGAAAGCAATT
RNA2     ATTTTGCCTTACTGGACACCTACCCAAACGAAAC-----GAAACGAAACGAACCGAACCGAAAAGAAAAGAAACGACAT
RNA3     --------------------------CACGAAAC-----GAAACGAAACGAACCGAAAAGAACAGAACCGAGCACTAAG
RNA A    ----AGCCTTACTGGACACCTACCCAAACGAAAC-----GAAACGAAACGAAAACAAACGAACAGAACAGAAAATAAAT
RNA B    ---------TACTGGACACCTACCGAAACGAAAA-----GAAACGAAACGAAAAGAAACGAAACGAAAATAAAGCAACC
RNA C    ---------AACTGGACACCTACCTAAAATAAAC-----GAAACGAAACGAAACGAACCGAAACGAAACGAAACTAAGT
RNA9     TTTGTATCTTACTGGACACCTACCGAAACGAAAACAACCGAAACGAAACGAAACGAAAACAAAAGAAATTTGGTCAGAC
```

B)

```
RNA1     TA------------------AT-GTTGGTCGTC-CTGTACGTT-CGTGTACAGGTTGGTG--TTA--TTCACTTACAGCGATAG-
RNA2     TA--GTTCATTA-AATTTTTATTGTTGGTCGTCTCATTCGGTT-CGCGAGTGAGTTGGTG--TTA-CACCAT-----GCGCGC--
RNA3     TA--GTTCATAATATTTTT-AACGTTGATCGTC-CC-GTGCGTGCGTGCATGGGTCTATGG------------------------
RNA A    TATTGTTTATTATGTTTTT-ATTGTTGATCGCC-CTTTGGATTTCGTCCAGGGGTCTTTGGGTTA--CCCATTGAGAGCGATA--
RNA B    TATTGTTTATTATGTTTTT-ATTGTTGATCGCC-CCTTGGATTTCGTCCAGGGGTCTTTGGGTTA--CCCATTGAGAGCGCTA--
RNA C    TAT-GTTCATTATGTTTTT-ATCGTTGGTTGCC-CTTTGAAGTTCGTTCAGAGGTCGTTGAGTTATCTCAAC-GAGAGCAACAGA
RNA D    TATTGTTTATTATGTTTTT ATTGTTGATCGCC-CTTTGGATTTCGTCCAGGGGGCTTTGGGTTT–CCCATTGAGAGCG------
```

Figure S1. Multiple nucleotide sequence alignments of A) the 5' non coding region (NCR) and B) the 3' NCR of the contig assembled using the sequence data of SRA SRX3209419 for the various genomic segments identified for Carya-associated jivivirus 1 (CaJV-1). Residues conserved in all RNA segments are highlighted in yellow.

A)

```
GaJV-2  P3    CFYDMCSLAGVEVPFGVTRVADLDEDAMSALAAKRQFKVLLRAYSSINDPRNHVELVRNDATNFKSYIRGPFDLEQDFYYLVCTSTLHFFNKGNEWSKFHFFS
GaJV-1  P9    CLARACGKAGVFVPYNIRRVADMSKSALTTLSAMKGFKVLVKAYSQ-KDKRNHIELIGNEAGNFLSKITFPESGGVDDHVLSVTTMQHFFNSSNQWASHKFYA
CaJV-1  P9    CFSLACRAAGVNVPPHVRSVRDMDDRDMVAMAAKP-FKVAFVVYAA-DDSRNHVELVPANATNALVHVKPVTSTSHHHRVFVCTTVQQFFNKGNQWAGYVFYA
CaJV-2  P9    CFARACRLAGVEVPGIATSVDQLRGDDVTKLSESLDFKVVVTCFGA-DDPRNHIDLVPSGTSNALVSVKPIDVIAQRHYVFACTTMQQFFNKGNRWYHTKFYA
```

B)

```
GaJV-1  P9    APGSIRAESV-SGGSANIGGGVSRPRKRVVGAKSGLMFGTAGVNKNYRTTGAILFNNAEELFQDSISRTYAQEIIEEVFDTWGVPMDQPEAATYAENLLWTFI
CaJV-1  P9    APESIVEDHVRSGGS-NVSGGVVRSRKRVIPARSTALDG--PVVKNYRTTNAVLFNNVGELFQDSISREYAQQIIEDLFETWGVPLDQPDAVKYAEDLLWSFL
CaJV-2  P9    PPESIVGDDVTGGGS-NVSGGVVRSRRRNISAR-ITAVG-VPVAKNYRTTGAVMFNNFDELFQDKVSRPFAQEIIEATFEAWGVPLDQPDAVKFAEDLLWSFI

              VAVTASNKADYNRTYDIPVRPIERNGESVTSVEADFSIFSRILEAKFGITRRQFSRGVADDLRNFLRHDENQALLPTLATRVGCEPLMAYLAFDGSTHCTGLT
              VSVTASNKADYNRVFDVPVKPIDRGSESVTEVEADFSKLSVLLASKYGLTRRQFARGVADDLRAFLKLEENQLILPQLATRIGCEPLLAYLAFDGSTHCSNMT
              VAVTASNKADYNRVYDIPVRAIKRGGETLNSVDADFAVFSKLLEFQFGVTRRQFARGVSDDLKDFLRREENQALLPTLATRVGCEPLLAYLAFDGSTHCTGLT

              TREAAFTRTLEQRNLFERDDENAAGASDRLMQGFSGSVRSVQPR*
              SRETTFTKTLESRNLFERDDVVAQGASDRLMQGMSGGVRAVSSR*
              TREVTFTRTLESRNLFERDDVLAQGSSDRLMQGVSGGVRSVSVR*
```

Figure S2. Multiple protein sequence alignments of A) the conserved domain between the P9 proteins of GaJV-1, CaJV-1 and CaJV-2 and the P3 protein of GaJV-2 (MN520750) and B) the C-terminal region of the P9 proteins of GaJV-1, CaJV-1 and CaJV-2. Residues conserved in all proteins are highlighted in yellow.

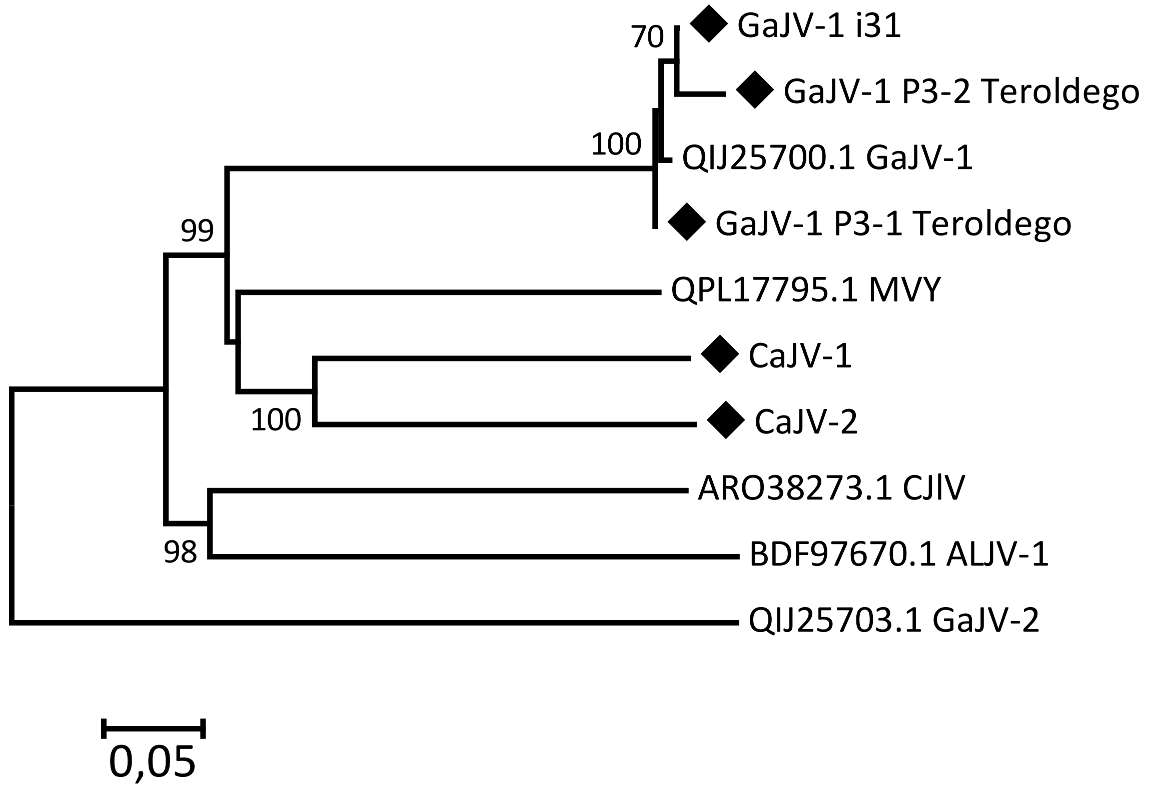

Figure S3. Phylogenetic tree reconstructed from the sequences of the P3 proteins of GaJV-1, CaJV-1, CaJV-2 and selected related agents. The tree was reconstructed using the neighbor-joining method and strict amino acid distances. Branch validity was evaluated by bootstrap analysis (1,000 replicates) and only bootstrap values >70% are shown. Sequences determined here are marked by a black diamond. The scale bar represents 5% aa divergence. GaJV-1 (or -2): grapevine-associated jivivirus 1 (or -2); CaJV-1 (or -2): Carya-associated jivivirus 1 (or -2); CJlV: Citrus jingmen-like virus; MVY; Mastic virus Y; AlJV-1: Aspergillus lentulus jivivirus 1.

```
                           ! !** * !  !!! !  !   !*       ! ! !  !    ! ***** *   !     *!  *! !** *       !!*  !  !          !
>GaJV-1                    GITRRQFSRGVADDLRNFLRHDENQALLPTLATRVGCEPLMAYLAFDGSTHCTGLTTREAAFTRTLEQRNLFERDDENAAGASDRLMQGFSGSVRSVQPR
>CaJV-1                    GLTRRQFARGVADDLRAFLKLEENQLILPQLATRIGCEPLLAYLAFDGSTHCSNMTSRETTFTKTLESRNLFERDDVVAQGASDRLMQGMSGGVRAVSSR
>CaJV-2                    GVTRRQFARGVSDDLKDFLRREENQALLPTLATRVGCEPLLAYLAFDGSTHCTGLTTREVTFTRTLESRNLFERDDVLAQGSSDRLMQGVSGGVRSVSVR
>Gleditsia sinensis        GLTRRQFARGVADDLRTYLKLPENQPLLPKLATRIGCEPLLACLAFDGSTHCTGLTTRETTFTKTLESRNLFERDDVVAQGASDRLMQGMSGGVRAVSSR
>Ginkgo biloba             GVTRRQFARAVADDLRAFLKKEENQAMLPVIATRVGCEPLLAYLAFDGSTHCTGMTTREAAFTKTLESRNLFEDDSLMGAGASDKLFQGF----------
>Bletilla striata          EVTRRRFARAIADDVRKFIMEPNNVRLREMLVTELGVKSEYAGLAFDGSTHCSGMSTNQVMFTKELERMNLFDEQKVRDKMASNQQLATAGFQVNARRGAYN
>Cenostigma pyramidale     GITRRRFSRGVANDLRDFLRHPENQFMLPELATRVGCDPQLAHLAFDGSTHCSGMTSREVAFTKTLESRNLFEREDVLAQGASGKLMQGVVE-SRSVVPR
>Selaginella lepidophylla  GVTRRQFARAVADDVRVYLMHVENVHLLPELARRAGCDPQMATLVFDGSTHCSGLTSQEITFTKLLQARNLFEDDSVLADDASARLMQGSVGGARSRVPR
>Ginkgo biloba             GVTRRQFARAVADDLRSFLKKEENQAMLPLIATRVGCEPLLAYLAFDGSTHCTGMTTREAAFTKTLESRNLFEDDSVMGVGASDRLFQGFSGGPRSVVPR
>Lumnitzera littorea       GVTRRNFARGVADDLRAYLNRTENQFVKTKSAARIYADPQYGDLSFDGSTGCTGMTTSQQRFTKLLEDRNLYERADVRADGASDILMQGIHGGSRSVVQR
>Ilex paraguariensis       GITRRNFARGVANDIRGYLNRPENQFVKSKSAERIYADPQYGSLSFDGSTGCLGLTSSERKFTKLLEDRNLYERADL----------------------
>Viola albida              GKTRRQFARAIADDQKNFLRHEENKPLLPRLATRVGCETLLAYLSFDGSTP-------------------------------------------------
>Tectona grandis           GVTRRQFARGMADDMRAYIKDPDNTHLRPKLADRAGCDPQMAELAFDGSTHCTGLTSAQLTFTRLLESRNLFEDDAVLSQGTSQTIMEGIHGHVRSKVQ-
>Viola orientalis          GITRRNFARGVADDLRSYLQREGNQFIVSRISQRVGADPQMGTLCFDGSTNCTGLTTSELTFTKVLEGRNLFERDDVIAEGASGRLFQGMSGGVRSVVPR
>Phalaenopsis aphrodite    GITRRNFSCGIADDQRRFLQRPENTHLLPVLATRVGCDPQFATLAFDGSTHCTGLTSREVGFTKTLKVRNLFESDEMLAPGASDKLLQGMPAGVRSVVPR
>Iris domestica            GITRRNFSCGIADDQRRFLQRPENTHLLPLLATRVGCDPQFATLAFDGSTHCTGMTSREVGFTKTLKVRNLFESDEMLAPGASDKLLQGMPAGVRSVVPR
>Persea americana          ----------------ITAAGEENQHILPTLATRIGCEPLMAYLAFDGSTHCDNLTTREVTFTKTLESRNLFERDDVLAQGSSDRLMQGMSGGVRSVAPR
>Mangifera indica          GVTRRNFARGAADFMRNFLSRPENQQVKVLAAQRVGADPQYGALCFDGSTGCSNLSSVHQKFCALLEGRNLYEREDLIAEGASDKLMEGIHGSVRSVAPR
>Mangifera indica          GITRRNFSRAISDDLREYLNRSENQFIKSKSAQRIGADPQFGALCFDGSTHCSNLTSSESTFTRYLESRNLYERADVIAEGASDALMQGLHGGVRSVVPR
>Cenostigma pyramidale     GISRRNFARGIADNLRAYLRRDENQHLKSLSAQRIGADPQMGDMCFDGSTGCTSLTSSEASFARLLEARNLYERDDVIAEGASDKLMQGMSGGVRSIVPR
>Aquilaria malaccensis     GVTRRQLARGLANDFRRFLKQEENKYLLPILATRIGCDPQFADLAFDGSTHCSGMTSREIAFTKILESRNLFEDEAVLAAGASDRLMNGVSTSRASKVPR
>Vriesea carinata          GVTRRQFARAVADDQHGYIRNEANKFLLPILATRIGCDPQYAHLAFDGSTHCSGMTSREMTFTRTLESRNLFEDESVLAAGASERLMDGPRVGVSSKVPR
>Bambusa oldhamii          GVTRRQFARAIADDMRTFIKDPENIHLLPRLASRVGCDPQMASLGFDGSTHCSNLTTAELSFTKLLENRNLFEDDSLLAEGASQRLMQGMSSGPRSIISR
>Sarcandra glabra          GVTRRQFARALADDQRDFLRLPDNKFLLPTLATRIGCDPQYAHLAFDGSTHCTNLTSREVSFTKTLESRNLFEDESILAAGASDTLMNGPRVGVSSKVPR
>Ardisia crenata           SVTRRQFARGIADALHRYLKQPENAHLLPNLATRVGCDNQLASFAFDGSTHCTGMTSMEMRFTRLLETRNLFEDDAVLANGASDRLMQGMGSGARSVQTR
>Juniperus ashei           GVTRRQFARAVADDLRDYLKHEENQYMLPILATRIGCESLLSDLAFDGSTHCTGLTTRQVAFTKTLESRNLFERDDVVAQGASERLMQGFSGGVRSIAPR
```

Figure S4. Multiple protein sequence alignments of the C-terminal conserved domain of the P9 proteins of GaJV-1, CaJV-1 and CaJV-2 and of proteins encoded by contigs identified by TBLASTN analysis in the transcriptome assembly of various plant species. Residues conserved in all proteins are highlighted in green and an asterisk above the alignment. Residues conserved in >80% of proteins are highlighted in yellow and a ! sign above the alignment. For the various plant species, the contigs used are: *Gleditsia sinensis* (GCKC01203879.1 and GCKC01201897.1); *Ginkgo biloba* (GHLL01078026.1 and GHLL01124537.1); *Bletilla striata* (GGUU01098164.1); *Cenostigma pyramidale* (GIYP01145516.1); *Selaginella lepidophylla* (GIMG01058596.1); *Lumnitzera littorea* (GGFO01008820.1); *Ilex paraguariensis* (GEWR01020459.1); *Viola albida* (GFWC01055794.1); *V. orientalis* (GFXR01028129.1); *Persea americana* (GHOF01010909.1 and GHOF01010909.1); *Tectona grandis* (GFGL01201602.1); *Phalaenopsis aphrodite* (JI811267.1); *Iris domestica* (GGPC01141670.1); *Mangifera indica* (HOG01045463.1 and GHOG01041455.1); *Cenostigma pyramidale* (GIYP01566055.1); *Aquilaria malaccensis* (GGSI01128087.1); *Vriesea carinata* (GHCB01019885.1); *Bambusa oldhamii* (GJPT01309189.1); *Sarcandra glabra* (GJAT01069010.1); *Ardisia crenata* (GJZC01035951.1) and *Juniperus ashei* (GEWT01000925.1).
